# KANK1 regulates the positioning of liprin-α1 and the spatial organization of insulin granule fusion in pancreatic β cells

**DOI:** 10.1016/j.jbc.2025.111036

**Published:** 2025-12-09

**Authors:** Kylie Deng, Kitty Sun, Hayley Webster, Belinda Yau, Thomas Loudovaris, Helen E. Thomas, Melkam A. Kebede, Peter Thorn

**Affiliations:** 1 School of Medical Sciences, Charles Perkins Centre, University of Sydney, Camperdown, Australia; 2 St Vincent’s Institute of Medical Research, Fitzroy, Australia

**Keywords:** β-cell, insulin, secretion, presynaptic scaffold, capillary, islet

## Abstract

In β cells, insulin granule fusion is enriched at the capillary interface, which targets insulin secretion directly into the bloodstream. The cues and molecular mechanisms used to target granule fusion to this area remain unknown. The capillary interface is characterized by local activation of focal adhesions and an enrichment of presynaptic scaffold proteins, including liprin-α1, the latter suggesting that a presynaptic-like mechanism might control the targeting of insulin granules to this region. Here, we show that the focal adhesion-associated adaptor protein KANK1 is locally enriched at the β-cell capillary interface and its knockdown disrupts the subcellular localization of liprin-α1. Moreover, KANK1 knockdown reduced glucose-induced insulin secretion and led to the mistargeting of insulin granule fusion. We provide evidence that KANK1 is a component in a complex that links the focal adhesion protein talin to liprin-β1, which in-turn anchors liprin-α1 through binding at its C-terminus. We conclude that the local activation of focal adhesions at the capillary interface provides the primary cue to orient the β-cell. The subsequent enrichment of KANK1 provides the molecular link between focal adhesion localization and the positioning of liprin-α1. Liprin-α1 is a key presynaptic scaffold protein, and although the mechanisms are not known, its local enrichment is likely to drive targeted insulin granule fusion.

In pancreatic β cells, insulin granule fusion is localized to the vascular face of β cells where they contact adjoining islet capillaries, thereby delivering the hormone directly into the bloodstream (1, 2). The mechanisms that localize granule fusion to this region are unknown, but accumulating evidence indicates this β-cell capillary interface has characteristics analogous to the neuronal presynaptic active zone (1, 2, 3, 4). Like the active zone, the capillary interface is characterized by an enrichment of presynaptic scaffold proteins, including liprin-α1, ELKS and RIM, and is also a region showing preferential Ca^2+^ entry and localized insulin granule exocytosis (1, 2, 3, 5). Furthermore, studies have shown that many of these scaffold proteins, including ELKS (3) and RIM (6), are functionally important for insulin secretion. However, unlike synapses in neurons, β cells lack a postsynaptic domain, which in neurons plays a crucial role in the precise alignment and positioning of presynaptic scaffold proteins (7). In the absence of a post-synaptic domain, how β cells orientate and position presynaptic machinery to the capillary interface is unknown.

One potential candidate for positioning presynaptic scaffold proteins in β cells is the extracellular matrix (ECM). In both mouse (8) and human (9) islets, the ECM is present as a basement membrane that is highly enriched around the islet capillaries. β cells make discrete points of contact with this capillary ECM and these contacts have been shown to define β-cell polarity (5, 10), and are essential for maintaining normal glucose-stimulated insulin secretion (11, 12, 13, 14, 15) and glucose sensitivity (1). Previous work has demonstrated that ECM, and consequent focal adhesion (FA) activation, is a key factor affecting the subcellular distribution of liprin-α1 in β cells *in vitro* (1, 5). Using β cells grown on alternating microprinted stripes of ECM and E-Cadherin, Gan *et al.* showed that liprin-α1 preferentially clustered on the ECM stripes compared to E-Cadherin (5). Moreover, liprin-α1 and ELKS were both found to be significantly enriched at the ECM/coverslip interface of β cells grown on ECM-coated coverslips, and this enrichment was lost in the absence of ECM (1), providing indirect evidence suggesting that ECM may be controlling the positioning of presynaptic scaffold proteins in β cells.

The molecular mechanisms that might link the β-cell synaptic scaffold proteins to the ECM are unclear. However, new insights have come from the non-synaptic roles of ELKS and liprin, where they play roles in cell adhesion, cell motility, and oncogenic signaling (16, 17). Work on these roles reveals ELKS and liprin form part of a cortical microtubule stabilization complex (CMSCs) that interacts with FAs and with the microtubule system (18, 19, 20, 21, 22). Excitingly, it has recently been shown that many components of the CMSCs, including the proteins KANK1 and Ll5β, are expressed in both mouse and human β cells (23). Immunostaining for KANK1 and Ll5β showed significant colocalization with the active zone proteins RIM and ELKS *in vitro* (23), supporting the idea that CMSCs might be present in β cells. However, these localization studies were performed in dispersed β cells and INS-1E cells, where capillary contacts and the normal environmental cues within an islet that drive cell orientation and regional specializations are lost (10). The subcellular organization of KANK1 and Ll5β in native β cells *in situ*, the functional roles of these proteins in insulin secretion, and the mechanisms by which this system might regulate targeted exocytosis remain undetermined.

Here, we use a pancreatic slice preparation that preserves the native structure of islets (1), to show that KANK1 and Ll5β are both locally enriched at the ECM interface of β cells. Knockdown of KANK1 impacts the subcellular positioning of liprin-α1 in β cells. Knockdown of KANK1 also reduced glucose-stimulated insulin secretion and impaired granule targeting. Finally, we show that liprin-β1, a known binding partner of KANK1, is present in β cells and locally enriched at the ECM interface and directly interacts with the C-terminus of liprin-α1. We propose a model where KANK1 links and positions presynaptic exocytotic machinery to FAs at the β-cell capillary interface *via* liprin-β1 to regulate targeted insulin granule fusion.

## Results

**Figure 1:**
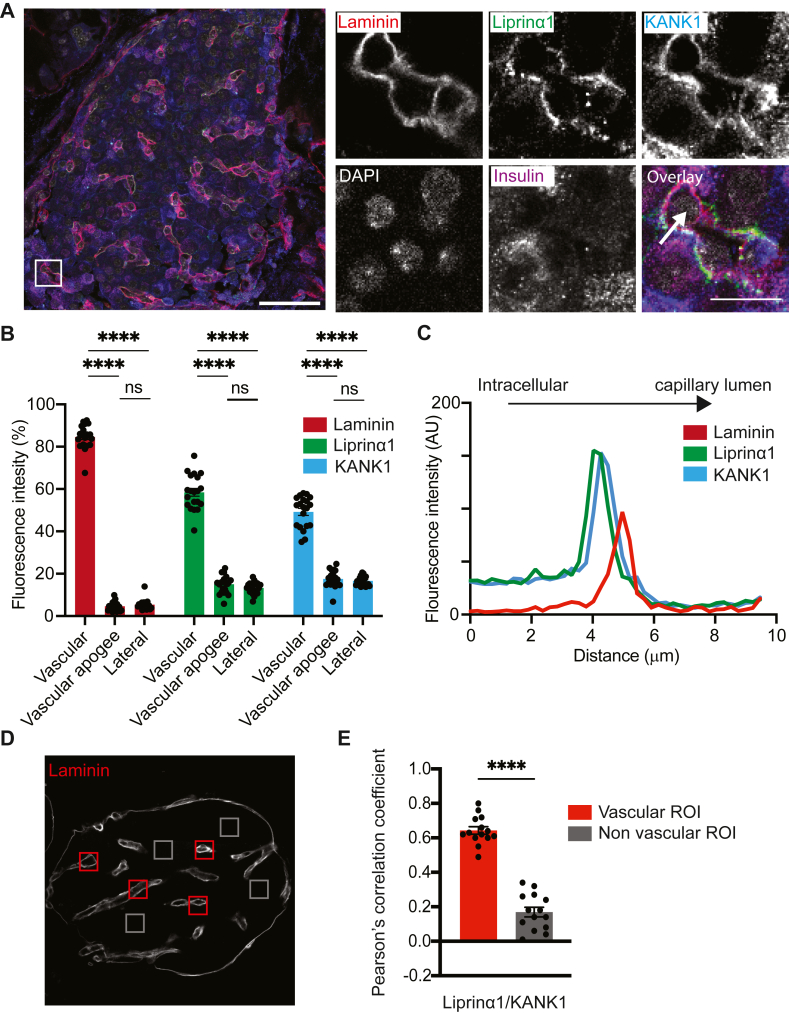
**The CMSC component KANK is enriched at the β-cell capillary interface.***A*, representative immunofluorescence of an islet within a mouse organotypic pancreatic slice. KANK1 (*blue*) co-locates with laminin (*red*) and liprin-α1 (*green*) at the capillary-interface of β cells (insulin, *purple*). Box indicates region for the zoomed images on the right. Scale bars 50 and 10 μm. *B*, fluorescence intensities of laminin, liprin-α1, and KANK at the vascular (capillary-interface) and vascular apogee (furthest away from the capillary) and the lateral regions of the β cells (20 cells, across 8 islets, from 3 animals) show significant enrichment (two-way ANOVA followed by Tukey multiple comparison) of all at the β-cell capillary interface. *C*, laminin, liprin-α1, and KANK intensities along a linescan drawn perpendicular to an islet capillary (see *arrow* on zoomed image in *A*) shows local liprin-α1 and KANK1 enrichment adjacent to that of laminin. *D* and *E*, comparison of regions of interest at the β-cell capillary interface with those away from the capillaries (14 ROIs, across 4 islets, from 3 animals) shows a positive Pearson’s correlation coefficient between liprin-α1 and KANK only at the capillary interface. (∗∗∗∗*p* < 0.001).

Insulin granule fusion principally occurs at the interface of β cells with islet capillaries (1) a region readily identified in pancreatic slices by immunostaining for capillary ECM proteins like laminin (1, 2). If proteins of the CMSC control the localization of presynaptic scaffold proteins (23) which in turn control the localization of granule fusion, we would expect evidence that they are present in this region. To test this, we used fixed mouse pancreas slices, where native structure is well maintained, and immunostained for laminin to identify the islet capillaries and insulin to identify β cells. The data show extensive islet capillary beds with liprin-α1 locally enriched in β cells specifically where the β cells interface with the capillary (Fig. 1*A*). Co-staining for KANK1 also showed enrichment at this capillary interface (Fig. 1*A*) with the overlay images indicating that liprin-α1 and KANK1 are colocated. To test for this, we measured the fluorescence intensity in regions of interest placed in regions of the β cells, at the vascular face, the vascular apogee (far away from the capillary) and along the lateral sides of the cells (see (10) for details of β-cell polarity). We used laminin as the capillary ECM protein to identify the vascular face of β cells since it is not found in the other cell regions (Fig. 1*B*) and show both liprin-α1 and KANK1 have a significant enrichment at the vascular face. Measuring fluorescence intensity along a linescan drawn perpendicular to the vascular face showed that liprin-α1 and KANK1 have overlapping peaks of fluorescence, and the laminin peak is offset, consistent with an extracellular location of laminin and intracellular co-localization for liprin-α1 and KANK1 (Fig. 1*C*).

The images of Figure 1*A*, indicate that liprin-α1 and KANK1 have both regions of overlap and regions where they are individually enriched. To quantify these distributions, we performed a Pearson’s correlation coefficient comparing regions of interest over the capillaries compared to regions away from the capillaries (Fig. 1, *D* and *E*). The results show a significant correlation between KANK1 and liprin-α1 is only found in the capillary regions. We conclude that a key CMSC protein, KANK1, is selectively enriched at the capillary interface of pancreatic β cells and is significantly colocalized with liprin α1.

**Figure 2:**
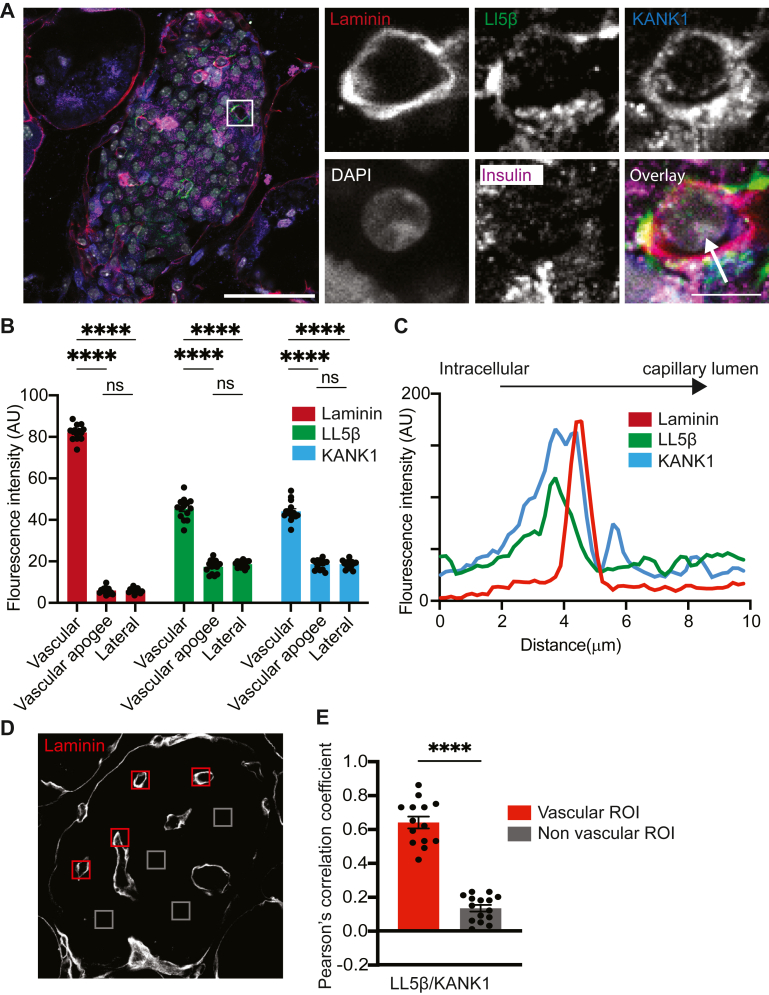
**The CMSC component LL5β is enriched at the β-cell capillary interface.***A*, representative immunofluorescence of an islet within a mouse organotypic pancreatic slice. LL5β (*green*) co-locates with KANK1 (*blue*) laminin (*red*) and at the capillary-interface of β cells (insulin, *purple*). Box indicates region for the zoomed images on the right. Scale bars 50 and 10 μm. *B*, fluorescence intensities of laminin, KANK1 and LL5β at the vascular (capillary-interface) and vascular apogee (furthest away from the capillary) and the lateral regions of the β cells (14 cells, across 5 islets, from 3 animals) show significant enrichment (two-way ANOVA followed by Tukey multiple comparison) of all at the β-cell capillary interface. *C*, A linescan of laminin, KANK1 and LL5β intensity through an islet capillary (see *arrow* on zoomed image in *A*) shows local KANK1 and LL5β enrichment adjacent to that of laminin. *D* and *E*, comparison of regions of interest at the β-cell capillary interface with those away from the capillaries 14 ROIs, across 4 islets, from 3 animals) shows a positive Pearson’s correlation coefficient between KANK1 and LL5β only at the capillary interface. (∗∗∗∗*p* < 0.001).

We next performed a similar study on another CMSC protein, LL5β and on talin, which is a focal adhesion protein that is part of the complex. Both LL5β and talin were specifically enriched at the capillary interface of β cells, as shown in the images (Figs. 2*A* and S1*A*) and in the histograms of the regional fluorescence intensity (Figs. 2*B* and S1*B*) and linescans of fluorescence intensity (Figs. 2*C* and S1*C*). For both LL5β and talin, we counter immunostained with KANK1 and showed regions of colocalization (Figs. 2*A*, and S1*A*), which was significant at the capillary interface region when compared to regions away from the capillaries (Figs. 2, *D* and *E* and S1, *D* and *E*).

We conclude that KANK1, LL5β and talin are all present and specifically enriched at the β-cell capillary interface. Furthermore, they co-localize with each other and with liprin-α1. This adds significant new evidence to support the hypothesis that CMSCs play a role the regional control of granule fusion.

Pancreatic slices are an excellent model to study β-cell function, but they are difficult to manipulate, for example, infection efficiency with the virus is low (1). Furthermore, the complex 3D organization of the β-cell capillary interface makes it difficult to directly study this area. A long-standing model to study the β-cell response to ECM contact is the culture of isolated β cells onto ECM substrate-coated coverslips (24). In the context of localization of granule fusion, we have used this model to show that culture of β cells on to laminin-coated coverslips forms focal adhesions at the β-cell ECM interface (1, 5) and leads to local enrichment of liprin-α1 and local targeting of insulin granule fusion to this region (5)—all consistent with the characteristics of the native β-cell capillary interface. Furthermore, focal adhesions are activated right across the contact area (25), which greatly enhances our ability to study this region. We therefore used the culture of β cells on to laminin-coated coverslips as a platform for further studies.

**Figure 3:**
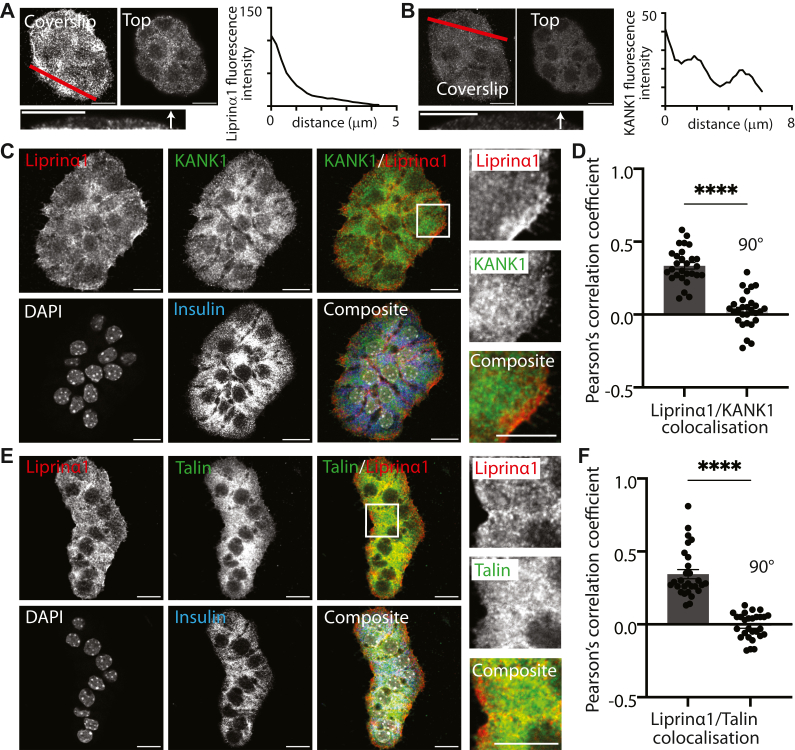
**KANK1, liprin-α1, and Talin all colocalize at the β-cell ECM interface in cells cultured on laminin-coated coverslips.** Serial Z sections of immunostained isolated islet cells cultured on laminin-coated coverslips show, in orthogonal sections, enrichment of (*A*) liprin-α1 and (*B*) KANK1 at the cell-ECM interface. *C*, single Z sections taken at the cell-ECM interface show that both liprin-α1 and KANK1 are spread across the whole of the footprint, with areas where they overlap, as shown (*D*) by a positive Pearson’s correlation coefficient. (28 ROIs across 3 mice). *E*, single Z sections taken at the cell-ECM interface show that both liprin-α1 and talin are also present across the footprint. The widespread distribution of talin reflects the formation of focal adhesions where the cells contact the coverslip, and in (*F*), Pearson’s correlation coefficient shows significant overlap (28 ROIs over 3 animals). ∗∗∗∗*p* < 0.0001 Error bars are mean ± SEM. Scale bars are 10 μm.

Isolated islet cells were cultured overnight on laminin-coated coverslips and then immunostained with insulin, used as a marker for β cells. Orthogonal sections through cell clusters showed enrichment of liprin-α1 (Fig. 3*A*) and KANK1 (Fig. 3*B*) at the coverslip interface, as expected. Single XY images, at the footprint where the cells contact the coverslip, show liprin-α1 and KANK1 spread out across most of the contact area (Fig. 3*C*). This reflects the activation of focal adhesions across the whole of this area (see (25)) and enables a more detailed examination of the overlapping areas of liprin-α1 and KANK1 than is possible in the slices. The analysis shows evidence for a positive Pearson’s correlation coefficient (Fig. 3*D*) with the images showing regions of overlap as well as regions selectively enriched in one protein compared with the other. Similar results were obtained when immunostaining for liprin-α1 and talin (Fig. 3, *E* and *F*), supporting the data in the slices (Fig. 1 and 2) to show that liprin-α1, KANK1 and talin are co-located.

**Figure 4:**
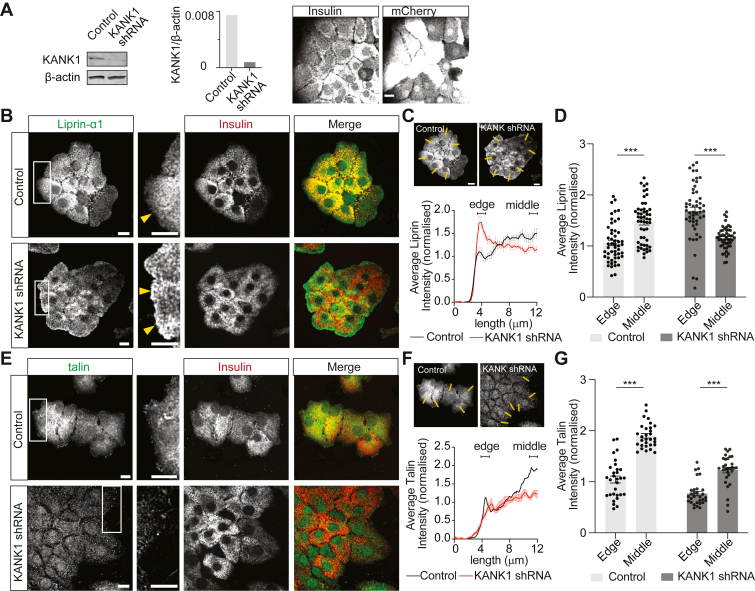
**KANK1 regulates the positioning of liprin-α1 in β cells.***A*, Western blot showing KANK1 protein expression in mouse islet cells infected with adenovirus containing either scrambled or KANK1 shRNA-mCherry. We normally obtained 80 to 100% infection efficiency in β cells indicated by the mCherry signal. Quantification of KANK1 protein expression normalized to β-actin is shown as a histogram. *B*, immunostaining of liprin-α1 in isolated mouse β cells cultured on laminin511-coated coverslips, imaged at the β-cell-laminin interface. In control cells, liprin-α1 (*green*) and insulin (*red*) are distributed across the β-cell-laminin interface. In KANK1 knockdown cells, liprin-α1 clusters at the periphery/edge of the cell clusters. *C*, 3 μm width linescans, perpendicular to the cell edge, were drawn from outside towards the inside of the cell clusters. Liprin-α1 fluorescence intensity along these linescans, normalized to average intensity over the entire line, shows a peak at the cell edge in KANK1 knockdown cells and not in the controls. *D*, a histogram showing liprin-α1 intensity at the cell edge *versus* middle (17 cells analysed from 3 animals; two-way ANOVA followed by Tukey multiple comparison, ∗∗∗*p* < 0.001). *D–F*, immunofluorescence staining of Talin shows a distribution across the β-cell-laminin interface in both control and KANK1 knockdown β cells (10 cells analyzed from 3 animals; two-way ANOVA followed by Tukey multiple comparison, ∗∗∗*p* < 0.001).

To further study the actions of the CMSC protein KANK1, we infected isolated mouse β cells with adenovirus co-expressing KANK1 shRNA and mCherry, and observed a significant decrease in KANK1 protein expression measured with Western blot (Fig. 4*A*). For subsequent imaging experiments, the mCherry signal was used to indicate successful infection (Fig. 4*A*).

We hypothesized that KANK1 might be a component controlling the positioning of presynaptic scaffold proteins, like liprin-α1. Using the model of culture of isolated cells onto laminin-coated coverslips, as in Figure 3 in control cells we observed a distribution of liprin-α1 across the entire cell footprint of β cells (Fig. 4*B*). In contrast, we observed a significant accumulation of liprin-α1 around the periphery/outer edges of the β-cell clusters after KANK1 knockdown (Fig. 4*B*), analyzed by taking linescans perpendicular to the cell edge (Fig. 4*C*) and measuring intensity at the edge compared to the inner/middle regions (Fig. 4*D*).

We also immunostained for the FA protein talin but did not observe any significant change in talin distribution after KANK1 knockdown (Fig. 4, *E*–*G*). This suggests that the alteration in liprin-α1 distribution is not due to a secondary effect of KANK1 knockdown on focal adhesion distribution. However, we also immunostained for a different FA protein, focal adhesion kinase (specifically the phosphorylated kinase, pFAK) and here (Fig. S1) we do see a significant effect of KANK1 knockdown on pFAK distribution. This suggests that KANK1 can disrupt some forms of FAs. The contrasting impact of KANK1 knockdown on pFAK and talin might reflect a specific effect on FA dynamics since these proteins are differentially recruited during FA maturation. Whatever the role of KANK1 in FAs our data demonstrate that KANK1 is involved in the localization of liprin-α1. Based on previous work, we therefore predicted that this disrupted positioning of liprin-α1 would lead to an impact on insulin secretion and specifically a mistargeting of insulin granule fusion.

**Figure 5:**
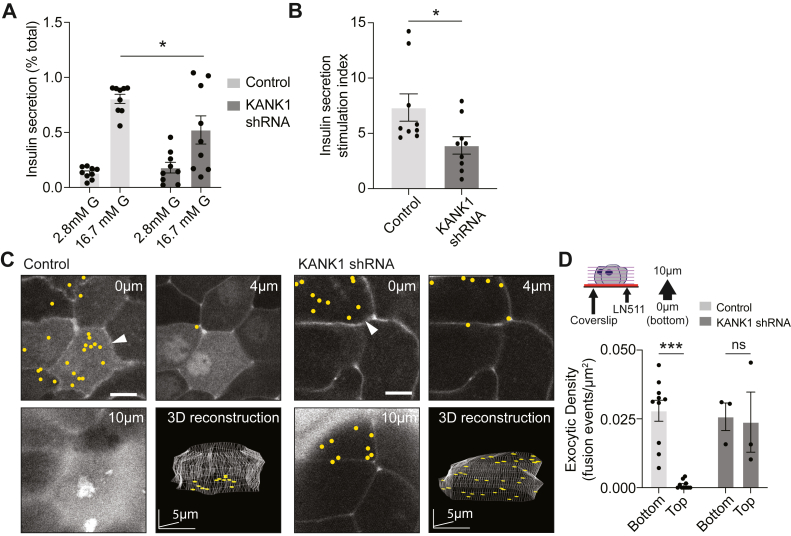
**Knockdown of KANK1 reduces GSIS and disrupts granule targeting.***A*, Glucose-stimulated insulin secretion, normalized to total cellular insulin content, in control and KANK1 knockdown mouse β cells. Insulin secretion was significantly reduced after KANK1 knockdown (n = 9 dispersed islet preparations from 3 animals; two-way ANOVA followed by Tukey multiple comparison, ∗: *p* < 0.05). *B*, histogram plotting the insulin stimulation index (fold increase in insulin secretion induced by a step increase in glucose concentration from 2.8 mM to 16.7 mM) in control and KANK1 knockdown β cells (Student’s *t* test, *p* < 0.05). *C*, isolated β cells cultured on laminin511(LN511)-coated coverslips were bathed in extracellular dye (sulforhodamine B) to visualize granule fusion events. Cells were stimulated with 16.7 mM glucose to induce granule fusion and recorded over 15 min in 3D (z stacks 2 μm apart) using two-photon microscopy. Exocytotic events are marked with yellow dots. *D*, a histogram of exocytotic density at the β-cell/laminin interface (*bottom*) *versus* regions of the cell away from the coverslip (*top*) shows a significant bias of granule fusion events towards the *bottom* in control but not KANK1 knockdown cells (n = 3–10 cells across 3 animals; two-way ANOVA followed by Tukey multiple comparison, ∗∗∗: *p* < 0.001).

We first tested whether knockdown of KANK1 in β cells affected bulk insulin secretion. Our data showed that with a stimulation of 16.7 mM glucose for 30 min, KANK1 knockdown decreased insulin secretion compared to the scrambled controls, both in measures of overall secretion (Fig. 5*A*) and as a reduction of the stimulation index (fold increase in insulin secretion over basal) (Fig. 5*B*). This data supports the idea that KANK1 is involved in the control of insulin secretion, but our hypothesis specifically proposes that KANK1 is a component in a mechanisms that localize granule fusion. To test for this, we employed 3D live-cell microscopy to map out granule fusion events (identified by the entry of extracellular dye) in β cells in response to glucose stimulation (see Low *et al.*, (2)). Consistent with our previous work (5), we showed that using isolated β cells cultured on ECM-coated coverslips and stimulated with 16.7 mM glucose, insulin granule fusion is targeted to the β-cell ECM interface (Fig. 5*C*). This targeting was quantified as a significant bias of the number of granule fusion events toward the β-cell ECM interface (bottom) compared to regions of the cell not in contact with ECM (top) (Fig. 5, *C* and *D*). In contrast, in KANK1 knockdown cells, granule fusion events were no longer targeted to the ECM interface and instead appeared evenly distributed across the entire cell membrane (Fig. 5*C*), quantified by measuring exocytotic density at the ECM interface (bottom) compared with cell membrane away from the coverslip (top) (Fig. 5, *C* and *D*).

The above results demonstrate that KANK1 plays an integral role in the spatial targeting of insulin granule exocytosis. The most likely mechanism that links KANK1 to the control of granule fusion is *via* its interaction with the presynaptic scaffold protein liprin-α1, and we next set out to test this possibility. Previous work in HeLa cells and the melanoma cell line SK-MEL has identified a potential link between KANK and liprin-α1 *via* the intermediary protein liprin-β1 (19, 26). KANK1 directly interacts with liprin-β1 to modulate FA shape in response to mechanical force in HeLa cells (27) and co-immunoprecipitates liprin β-1 in SK-MEL cells (26). Furthermore, liprin-βs are well known to hetero-oligomerize with liprin-αs (28).

**Figure 6:**
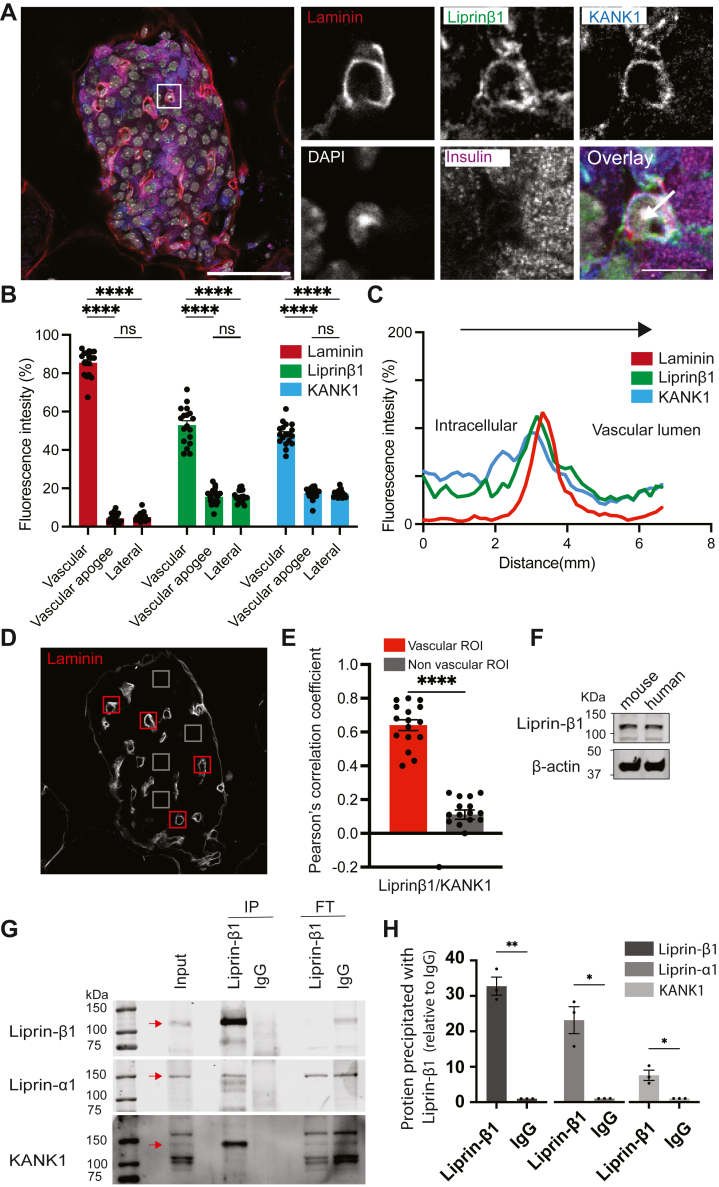
**Liprin-β1 is present in β cells, colocates with KANK1 and interacts with KANK1 and liprin-α1.***A*, representative immunofluorescence of an islet within a mouse organotypic pancreatic slice. Liprin-β1 (*green*) co-locates with laminin (*red*) and KANK1 (*blue*) at the capillary-interface of β cells (insulin, *purple*). Box indicates region for the zoomed images on the right. Scale bars 50 and 10 μm. *B*, fluorescence intensities of laminin, liprin-β1 and KANK1 at the vascular (capillary-interface) and vascular apogee (furthest away from the capillary) and the lateral regions of the β cells show significant enrichment of all at the β-cell capillary interface. (11 cells across 3 animals analyzed, two-way ANOVA followed by Tukey multiple comparison). *C*, A linescan of laminin, liprin-β1 and KANK1 intensity through an islet capillary (see arrow on zoomed image in *A*) shows local liprin-β1 and KANK1 enrichment adjacent to that of laminin. *D* and *E*, comparison of regions of interest at the β-cell capillary interface with those away from the capillaries 12 ROIs, across 4 islets, from 3 animals) shows a positive Pearson’s correlation coefficient between liprin-β1 and KANK1 only at the capillary interface. (∗∗∗∗*p* < 0.001). *F*, expression of liprin-β1 was confirmed by western blotting in both mouse and human islets. *G* and *H*, in the MIN6 β-cell line immunoprecipitation with anti-liprin-β1 antibody showed significant interactions with liprin-α1 and KANK1 as relative to IgG control (Student *t* test ∗*p* < 0.05 for both, n = 3, see Fig. S6 for original blots.

The presence of liprin-β1 has not been shown in β cells before, and therefore we used Western blot to demonstrate protein expression of liprin-β1 in mouse and human islets (Fig. 6*F*) and immunostaining in mouse pancreatic slices (Fig. 6, *A*–*E*). Using immunostaining in pancreatic slices, we examined the subcellular localization of liprin-β1. Our results demonstrated that like liprin-α1 (2) and KANK1, liprin-β1 is enriched at the β-cell capillary interface (Fig. 6, *A*–*C*). We counterstained with KANK1 and show that both liprin-β1 and KANK1 are enriched at the capillary interface (Fig. 6*B*) and their distributions overlap along a linescan drawn perpendicular to the capillary (Fig. 6*C*). A Pearson’s correlation coefficient analysis comparing regions of interest over the capillaries compared to regions away from the capillaries (Fig. 6, *D* and *E*) show significant correlation between KANK1 and liprin-α1 is only found in the capillary regions. To identify a possible molecular link, we used the liprin-β1 antibody in immunoprecipitation experiments and showed specific enrichment of both KANK1 and liprin-α1 (Fig. 6, *G* and *H*). Together, this data indicate that liprin-β1 forms part of the β-cell secretory complex and acts as the link coupling liprin-α1 to KANK1.

**Figure 7:**
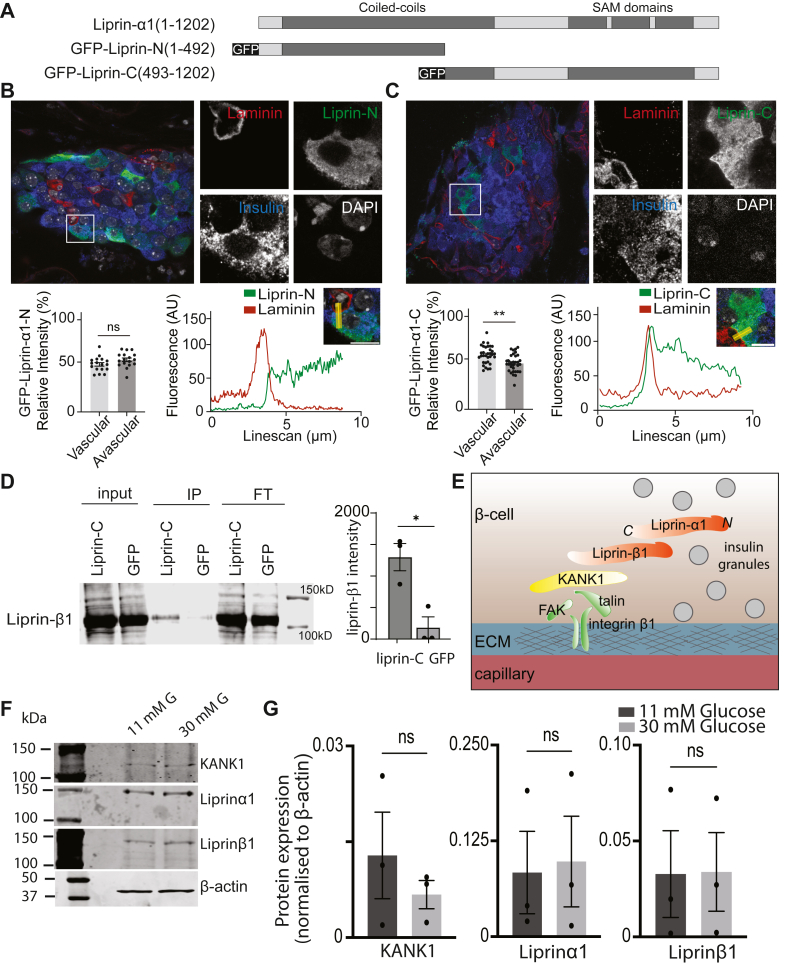
**Liprin-α1 localises to the ECM-interface *via* its C-terminus and interactions with liprin-β1.***A*, schematic of the domain organisation of liprin-α1 and the GFP-tagged truncated mutants generated. *B* and *C*, pancreatic slices were infected with adenovirus encoding the GFP-Liprin-N or GFP-Liprin-C mutants and immunostained. Relative GFP intensities at the vascular and avascular regions were plotted as histograms (≥17 cells across 3 animals analyzed), showing GFP-Liprin-C but not GFP-Liprin-N enrichment at the vascular face. This enrichment is also demonstrated with linescans. *D*, MIN6 cells expressing either GFP (control) or GFP-Liprin-C were lysed for immunoprecipitation with anti-GFP nanobody conjugated beads. Immunoprecipitates were analyzed by immuno-blotting against anti-liprin-β1 antibodies, showing a significant interaction between liprin-C and liprin-β1 (Student paired *t* test, *p* < 0.05, see Fig. S7 for original blots). *E*, cartoon model of the β-cell/capillary interface. KANK1 anchors or positions liprin-α1 to FAs/the ECM interface *via* liprin-β1, which interacts with the C-terminus of liprin-α1, to facilitate targeted insulin exocytosis into the islet capillaries. *F*, culture of the isolated islet cells for 3 days in 30 mM glucose had no significant effect on the expression of KANK1, liprin-α1 or liprin-β1 compared to culture in 11 mM glucose, as measured and quantified with Western blot.

To understand the molecular basis of this coupling, we generated liprin-α1 truncation mutants that dissected the two major domains of liprin-α1. Liprin-αs consist of a coiled-coil domain in the N-terminus known to interact with other presynaptic scaffold proteins, including ELKS (29) and RIM (30). At its C-terminus, liprin-α contains three consecutive sterile-alpha-motif (SAM) domains (28). Previous *in vitro* binding assays have demonstrated that the C-terminal region of liprin-α1 contains the binding domain for liprin-β1 (28) (Fig. 7*A*). Examination of the subcellular localization of the truncated liprin-α1 mutants in β cells *in situ* showed a local enrichment of liprin-C mutant, but not liprin-N mutant, at the β-cell capillary interface (Fig. 7, *B* and *C*). These results indicate that the liprin-α1 C-terminus is required for its vascular face positioning and supports the idea that the liprin-C mutant might be interacting with liprin-β1 to position/link it to KANK1 and focal adhesions. Next, we tested for this interaction using coimmunoprecipitation and confirmed an interaction between liprin-C mutant and liprin-β1 in MIN6 cells (Fig. 7*D*), supporting a model where liprin-α1 is anchored/positioned to the ECM-interface *via* KANK1 and liprin-β1 to control targeted insulin secretion to the capillaries (Fig. 7*E*).

Given this new evidence showing the involvement of KANK1 in a complex that regulates secretion and the targeting of insulin granule fusion we wanted to test if there was any relationship to disease. We used a glucotoxicity model by culturing the cells for 3 days in very high glucose concentrations of 30 mM and then measured expression of KANK1, liprin-α1 and liprin-β1 using Western blot. In all cases, we observed no significant change in expression (Fig. 7, *F* and *G*). We note that although protein expression is not changed, *in vivo*, the islet capillary structure does change, and this might impact this complex (31).

## Discussion

Our findings demonstrate a role for KANK1 in the spatial regulation of insulin exocytosis by coupling presynaptic secretory machinery to FAs at the β-cell capillary interface. We show that KANK1 is locally enriched at the capillary interface and colocalizes with liprin-α1, liprin-β1 and talin. We demonstrate that KANK1 knockdown disrupts the subcellular localization of liprin-α1 in β cells and impairs glucose-stimulated insulin secretion. Importantly, we show that this deficit in secretion includes a mistargeting of glucose-induced insulin granule fusion. We provide evidence for a complex where KANK1 interacts with liprin-β and talin to localize liprin-α1 at the β-cell-capillary interface. Our evidence lends further support to a model where three key protein complexes, FAs, CMSCs and presynaptic scaffolds, act together at the capillary interface to locally control insulin granule fusion.

The first work to identify KANK1 in β cells was by Noordstra *et al.* (23). Using immunostaining in dispersed human pancreatic islets and the rat INS-1E cell line, they demonstrated that KANK1 colocalized with the AZ proteins ELKS and RIM *in vitro* (23). However, this work is based on isolated β cells lacking capillary contacts and the normal intra-islet environmental cues, which are known to profoundly influence the structural organization of β cells and promote a distinct subcellular polarization that spatially segregates cell functions (5). We have previously shown using pancreatic slices, which preserve native islet structure that AZ proteins liprin, ELKS, RIM and piccolo are enriched at the β-cell-ECM interface where insulin secretion is targeted (1, 2). Here we demonstrate that KANK1 is also locally enriched at the ECM-interface of β cells *in situ* (Fig. 1), consistent with previous work linking KANK1 to focal adhesions (25) which are exclusively present at the β-cell-ECM interface (1, 5). Our identification of the polarized expression of KANK1 strongly supports a mechanism where it is involved in the spatial positioning of AZ proteins and, in turn the localized regulation of insulin granule exocytosis in β cells.

In neurons, AZ proteins are anchored to the presynaptic membrane *via* an array of trans-synaptic cues and cell adhesions molecules including neurexins and Leukocyte common Antigen-Related Receptor (LAR) (7). To fulfill this positioning/anchoring role, molecules must: 1. interact with and localize to the target plasma membrane, and 2. directly or indirectly bind the AZ proteins. Our results here, together with previous studies, indicate that in β cells KANK1 fulfills both these requirements and provides a molecular pathway linking liprin-α1 to the region of ECM enrichment at the capillary interface (Fig. 7*E*). KANK1 directly binds the focal adhesion protein talin (25) and is locally enriched at the capillary interface (Fig. 1). Consistent with previous findings (26, 27) we show (Fig. 6,*G*) that KANK1 binds liprin-β1, which we then demonstrate interacts with the C-terminus of liprin-α1 (Fig. 7*D*).

As in synapses, we observe redundancy and anticipate that this is not the only mechanism contributing to the positioning of liprin-α1. β cells do express both neurexins (32, 33) and LAR (unpublished data), although their subcellular distribution *in situ* has yet to be determined. Furthermore, we show that the membrane-associated protein Ll5β is also locally enriched at the ECM interface (Fig. 2). Ll5β binds the AZ protein ELKS and interacts with phosphatidylinositol-3,4,5-trisphosphate (PIP3) at the leading edge of migrating cells (18), therefore making it another plausible candidate for positioning AZ secretory machinery to the ECM interface. Clearly, more work is needed to elucidate this ECM-Ll5β-ELKS pathway in β cells and determine how these mechanisms might be acting together, whether redundantly or synergistically, to position liprin-α1 and other presynaptic scaffold proteins to the ECM-interface.

We have previously demonstrated that culturing β cells on ECM proteins *in vitro* models key aspects of the native β-cell capillary interface, such as targeted granule fusion (5) and local enrichment of presynaptic scaffold proteins to the β-cell-ECM interface (1). Here, we use this *in vitro* system to specifically test the effect of KANK1 knockdown on granule targeting, and our live cell granule fusion assay (Fig. 5) now provides direct evidence that KANK1 plays a critical role in the targeting of insulin exocytosis to the β-cell-ECM interface. We note that we do not expect this model to fully recapitulate all aspects of the native 3D islet environment; this *in vitro* system does allow us to manipulate and test in detail specific components contributing to targeted exocytosis.

Finally, we show that glucotoxicity does not affect expression levels of KANK1 (or liprin-α1, or liprin-β1), suggesting that it is not directly impacted in this model of a disease state. However, given the significant impact of diabetes on the islet capillary structure, it could be that the complex as a whole is affected, and this, in turn, could be a component in the alterations of insulin secretion seen as the disease progresses (31).

In summary, our results demonstrate that KANK1 positions and links liprin-α1 to FAs in β cells to regulate targeted insulin exocytosis to the β-cell/capillary-interface.

## Experimental procedures

### Mice

Male C57BL/6J mice were purchased from Australian BioResources (Moss Vale) and housed at the Charles Perkins Centre Laboratory Animal Services facility on a 12 h light/dark cycle in standard home cages in groups of up to five mice. All mice were given access to water and chow (7% simple sugars, 3% fat, 50% polysaccharide, 15% protein (wt/wt), energy 3.5 kcal/g) *ad libitum*. Mice (8–12 weeks old) were humanely killed according to local animal ethics procedures (approved by the University of Sydney Research Integrity and Ethics Administration Committee, project #2023/2300).

### Mouse islet isolation

Mouse islets were isolated from pancreatic tissue using a standard collagenase enzymatic digestion (34). Briefly, a 2 ml volume of 0.5 U/ml Liberase (TL Research grade, Roche) diluted in unsupplemented RPMI-1640 (Gibco) was injected into the pancreatic duct. The inflated pancreas was then dissected and placed into a 37 °C water bath for 14 min. Islets were separated from the resulting tissue using a Histopaque (Histopaque 1119 and 1077, Life Technologies) density gradient and then hand-picked in Hank’s buffered saline solution (Life Technologies). Isolated islets were cultured for 16 h (37 °C, 95/5% air/CO2) in RPMI-1640 (Gibco) supplemented with 10% fetal bovine serum (Gibco) and 1% penicillin-streptomycin (Invitrogen) before islet dispersion.

### Islet cell dispersion

Glass coverslips were coated with 5 μg/ml Laminin 511 (Biolamina) overnight at 4 °C. Prior to islet cell seeding, coverslips were briefly rinsed in phosphate-buffered saline (PBS). Islets cells were digested with TrypLE Express (Gibco) in a 37 °C water bath for 4.5 min. Islet cells were resuspended in RPMI-1640 (Gibco) supplemented with 10% fetal bovine serum (Gibco) and 1% penicillin-streptomycin (Invitrogen) and seeded, at high density, onto laminin-coated coverslips.

### Pancreatic slices

Pancreas sectioning was performed as described by Huang *et al.* (35). Briefly, 1.5 ml of warm (42 °C) 1% low melting point agarose (UltraPure LMP, Invitrogen) in extracellular slice medium (ECSM, 125 mM NaCl, 2.5 mM KCl, 1.25 mM NaH2PO4, 26 mM NaHCO3, 2 mM sodium pyruvate, 0.25 mM ascorbic acid, 2 mM myo-inositol, 1 mM MgCl2, 2 mM CaCl2, 6 mM lactic acid and 6 mM glucose at pH 7.4) was injected into the pancreatic duct. The inflated pancreas was dissected and immediately cooled by immersion in ice-cold ECSM. Pancreas tissue was then embedded in 2% agarose in 6- to 10-mm cubes and sectioned (200 μm thick) with a vibratome. Pancreatic slices were immediately fixed with 4% paraformaldehyde in PBS for 15 min at room temperature.

### Cell lines

MIN6 cells, purchased from AddexBio, were cultured in DMEM (Gibco) supplemented with 15% FBS (Gibco), 1% penicillin-streptomycin (Invitrogen), and 0.05 mM 2-Mercaptoethanol under standard culture conditions (37 °C, 95/5% air/CO2). Cells were regularly passaged upon reaching 80% confluency using TrypLE Express for 5 min at 37 °C. All cells used in this study were with passages <20.

### Human islets

Cadaveric human donor islets were obtained from St Vincent’s Institute, Melbourne. Informed consent was acquired, and the study was approved by the Human Research Ethics Committee at the University of Sydney (Project #2018/862). Islets were cultured under standard culture conditions (37 °C, 95/5% air/CO2) in RPMI-1640 (Gibco) supplemented with 10% fetal bovine serum (Gibco) and 1% penicillin-streptomycin (Invitrogen) before snap-freezing using dry ice.

**Table:** 

Donor	Age	Sex	Source
Islet preparation: 28 August 2023	53	F	St Vincent’s Institute, Tom Mandel Islet Transplant Program
Islet preparation: 12 December 2022	48	F	St Vincent’s Institute, Tom Mandel Islet Transplant Program
Islet preparation: 14 September 2022	56	M	St Vincent’s Institute, Tom Mandel Islet Transplant Program

### Adenovirus infection

All adenoviruses were purchased custom-made from Vector Biolabs. Mouse β cells, pancreatic slices or MIN6 cells were infected with adenovirus containing mCherry-(mouse) KANK1 shRNA, scrambled shRNA, GFP-(mouse) liprin-α1 aa1-492, GFP-(mouse) liprin-α1 aa493 to 1202 and GFP, as indicated (Fig. 2, 4days-f). Cells or slices were incubated with the virus for 24 h in their respective culture medium, washed twice and cultured for an additional 48 h before experiments.

### Immunofluorescence

All samples for immunofluorescence were fixed with 4% paraformaldehyde in PBS for 15 min at room temperature. Immunofluorescence was performed as described by Meneghel-Rozzo *et al.* (36). Pancreatic slices and dispersed β cells were incubated in blocking buffer (3% BSA, 3% donkey serum, 0.3% Triton X-100; 1 h for dispersed cells, 4 h for pancreatic slices) at room temperature. Primary antibody incubation was performed overnight at 4 °C, followed by secondary antibody incubation at room temperature (1 h for dispersed cells, 4 h for pancreatic slices). All primary and secondary antibodies were used at a 1:200 dilution: Anti-beta1 laminin (Thermo Fisher Scientific, MA5-14657), Anti-Insulin (Dako, A0564), Anti-KANK1 (Sigma-Aldrich, HPA056090), Anti-Ll5β/PHLDB2 (Abcam, ab202350), anti-E-cadherin (BD Transduction Laboratory, 610,181), Anti-liprin alpha1 (Proteintech, 14175-1-AP), Anti-Phospho-FAK (Cell Signalling Tech, 8556S), Anti-talin (Sigma-Aldrich, T3287), Anti-liprin beta1/Ppfibp1 (Thermo Fisher Scientific, PA5-51663), Alexa Fluor 546 Donkey anti-rabbit (Thermo Fisher Scientific, A10040), Alexa Fluor 488 goat anti-guinea pig (Thermo Fisher Scientific, A11073), Alexa Fluor 546 Donkey anti-mouse (Thermo Fisher Scientific, A10036), Alexa Fluor 633 Goat anti-rat (Thermo Fisher Scientific, A21094). DAPI (Sigma, 100 ng/ml final concentration) was added during the secondary antibody incubation. Samples were mounted using ProLong Diamond Antifade Mountant and imaged on a Leica SP8 confocal microscope with a 40× water or 63× oil immersion objective.

### Live-cell imaging

3D Live-cell multiphoton imaging was performed on a custom-made Olympus two-photon microscope with a 60× oil immersion objective (NA 1.42, Olympus). Excitation was at 850 nm, and fluorescence emission was detected at 550 to 650 nm with a frame rate of 6 Hz and a resolution of 10 pixels/μm. 3D images were collected at a frame rate of 6 Hz with Z sections 2 μm apart. For granule fusion assays, cells were incubated in 2.8 mM glucose Krebs-Ringer bicarbonate HEPES buffer (KRBH, 120 mM NaCl, 4.56 mM KCl, 1.2 mM KH2PO4, 1.2 mM MgSO4, 15 mM NaHCO3, 10 mM HEPES, 2.5 mM CaCl2 and 0.2% BSA, pH 7.4) containing extracellular dye (SRB, 8 mM). Cells were stimulated with 16.7 mM glucose KRBH containing 8 mM SRB, and exocytotic events were recorded as the entry of SRB into each fusing granule upon stimulation (5). Images were analyzed using FIJI ImageJ and MetaMorph (Molecular Devices) software. 3D projections were created using IMOD (The Regents of the University of Colorado).

### Glucose-stimulated insulin secretion assay and Homogeneous Time Resolved fluorescence insulin assay

A glucose-stimulated insulin secretion assay was performed as described in Jevon *et al.* (1). Briefly, cells were incubated in 2.8 mM glucose KRBH for 1 h (pre-basal wash), followed by 2.8 mM glucose KRBH for 30 min (basal recording), then 16.7 mM KRBH for 30 min (stimulated recording). All media and cells were kept at 37 °C and 5% CO2 for the duration of the assay. Cells were lysed by sonication in ice-cold lysis buffer (1% NP-40, 300 mM NaCl, 50 mM Tris–HCl, pH 7.4, protease inhibitor cocktail tablet). Insulin content was measured in all samples and lysates by HTRF (Insulin Ultra-Sensitive Detection Kit, Revvity) according to the manufacturer’s instructions.

### Immunoprecipitation

MIN6 cells expressing GFP (control) or GFP-(mouse) liprin-α1 aa493 to 1202 were lysed by sonication in ice-cold lysis buffer (1% NP40, 10% glycerol, 137 mM NaCl, 25 mM Tris, 1 X cOmplete Protease Inhibitor Cocktail, 1 X PhosStop phosphatase inhibitor, pH 7.4). Sonicated cell lysates were clarified by centrifugation at 18000*g* for 10 min at 4 °C and the supernatant was collected. Clarified cell lysates were incubated with GFP-Trap beads (25 μl bead slurry/4 mg protein) at 4 °C for 1 h with rotation. Beads were pelleted and washed twice with wash buffer (0.05% NP40, 10% glycerol, 137 mM NaCl, 25 mM Tris, pH 7.4) and once with PBS. Bound proteins were eluted by boiling the beads in 4% SDC, 0.1 mM Tris-HCl, pH 8.0, for 10 min at 65 °C with shaking. Eluted samples were analyzed by Western blot. IRDye secondary antibodies were employed and visualized by a Licor Odyssey CLx system.

### Statistical analyses

All data are presented as mean ± S.E.M. Statistical analyses were performed using Microsoft Excel and GraphPad Prism. The analysis included two-way ANOVA followed by Tukey multiple comparisons to test for the effects of two independent variables across the datasets and unpaired Student t tests to compare across two datasets with assumed normal distributions.

## Data availability

All data needed to evaluate the conclusions in the paper are present in the paper and/or the Supplementary Materials.

## Supporting information

This article contains supporting information.

## Conflict of interest

The authors declare no conflict of interest related to the content of this study.
