# Supplementary material for: KANK1 regulates the positioning of liprin-α1 and the spatial organization of insulin granule fusion in pancreatic β cells
Source: J Biol Chem. 2025 Dec 9;302(1):111036. doi: 10.1016/j.jbc.2025.111036 (PMC12803835; doi:10.1016/j.jbc.2025.111036)
Supplement: Supplemental legend [file mmc1.docx]

**Supplementary Figure Legends**

**Figure S1 The focal adhesion component talin is enriched with KANK1 at the β-cell capillary interface**

(A) Representative immunofluorescence of an islet within a mouse organotypic pancreatic slice. Talin (green) co-locates with laminin (red) and KANK1 (blue) at the capillary-interface of β cells (insulin, purple). Box indicates region for the zoomed images on the right. Scale bars 50 and 10 μm. (B) Fluorescence intensities of laminin, talin and KANK1 at the vascular (capillary-interface) and vascular apogee (furthest away from the capillary) and the lateral regions of the β cells (16 cells, across 6 islets, from 3 animals) show significant enrichment (two-way ANOVA followed by Tukey multiple comparison) of all at the β-cell capillary interface. (C) A linescan of laminin, talin and KANK1 intensity through an islet capillary (see arrow on zoomed image in A) shows local talin and KANK1 enrichment adjacent to that of laminin. (D, E) comparison of regions of interest at the β-cell capillary interface with those away from the capillaries 12 ROIs, across 4 islets, from 3 animals) shows a positive Pearson’s correlation coefficient between talin and KANK1 only at the capillary interface. (**** p<0.001).

**Figure S2. KANK1 knock-down redistributes the focal adhesion protein, pFAK**

(A) Immunostaining of phospho-focal adhesion kinase (pFAK) in isolated mouse β cells cultured on laminin511-coated coverslips, imaged at the β-cell-laminin interface. In control cells, pFAK (green) and insulin (red) are distributed across the β-cell-laminin interface. In KANK1 knockdown cells, pFAK clusters at the periphery/edge of the cell clusters. (C) 3 µm width linescans, perpendicular to the cell edge, were drawn from outside towards the inside of the cell clusters. pFAK fluorescence intensity along these linescans, normalized to average intensity over the entire line, shows a peak at the cell edge in KANK1 knockdown cells and not in the controls. (D) A histogram showing pFAK intensity at the cell edge versus middle (≥10 cells analysed from 3 animals; two-way ANOVA followed by Tukey multiple comparison, *** p<0.001). (G-I)

**Figure S3. Original blots of immunoprecipitation experiments with liprin β1**

**Figure S4. Original blots of immunoprecipitation experiments with GFP-Liprin C**
