# Supplementary figures and images for: KANK1 regulates the positioning of liprin-α1 and the spatial organization of insulin granule fusion in pancreatic β cells
Source: J Biol Chem. 2025 Dec 9;302(1):111036. doi: 10.1016/j.jbc.2025.111036 (PMC12803835; doi:10.1016/j.jbc.2025.111036)

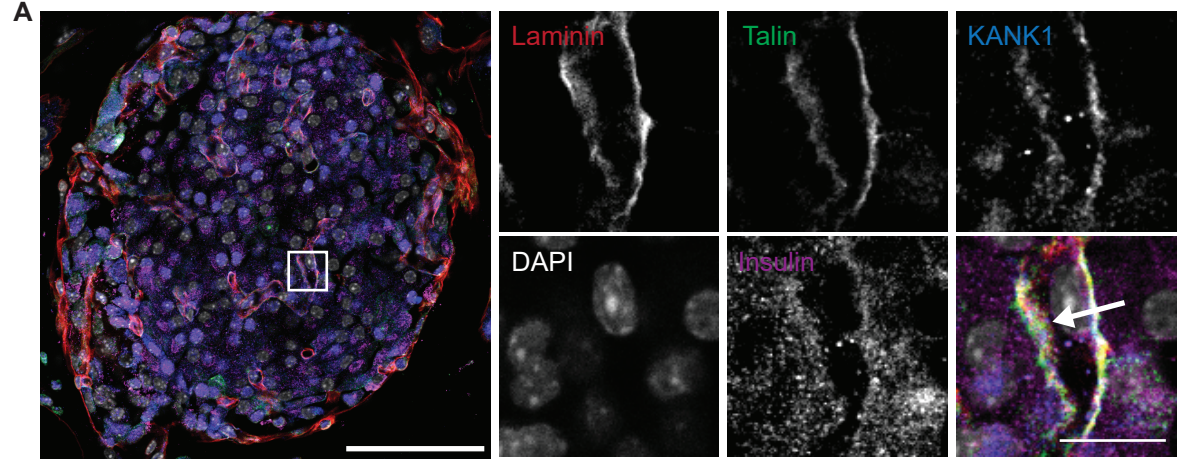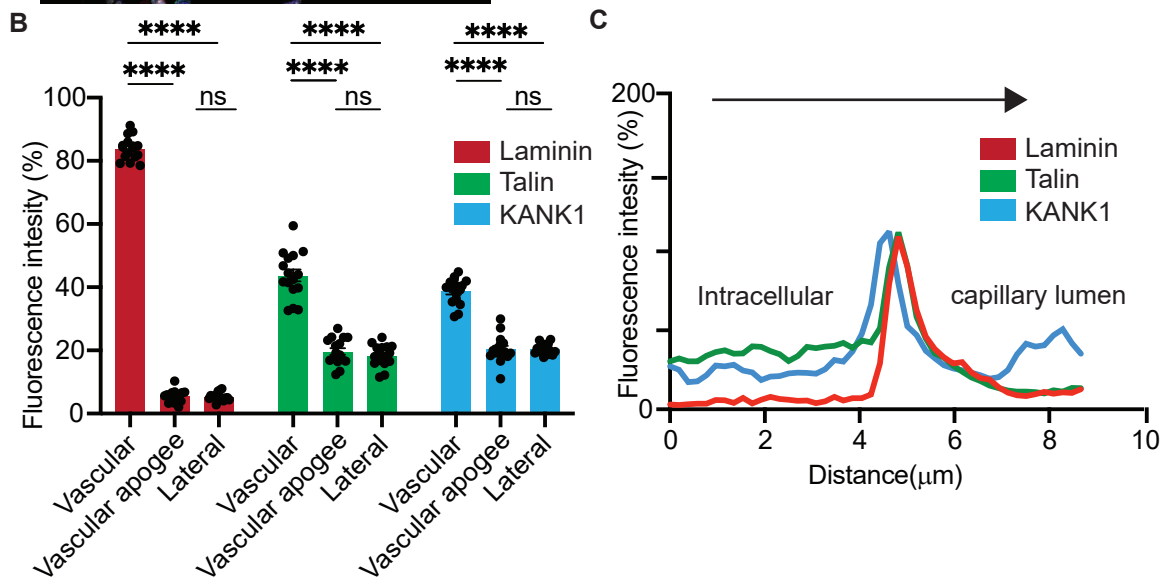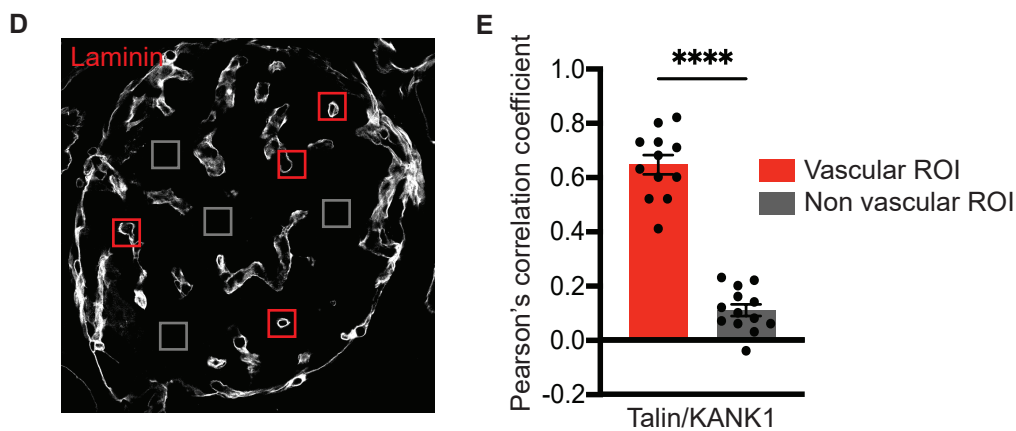

Supplement: Figure S1 [file mmc2.pdf]

**A**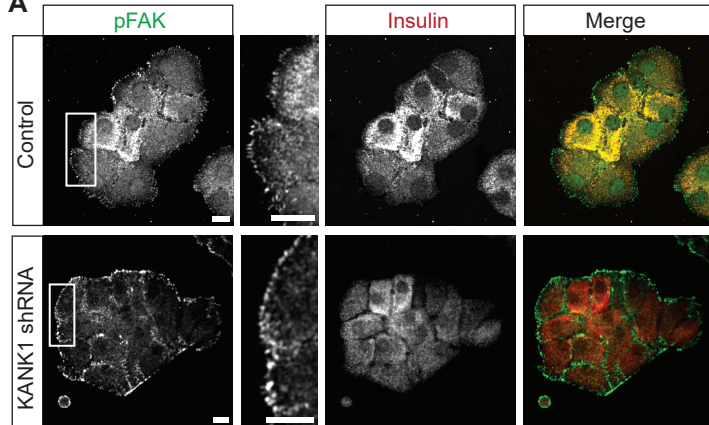**B**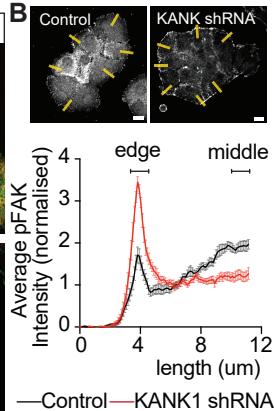**C**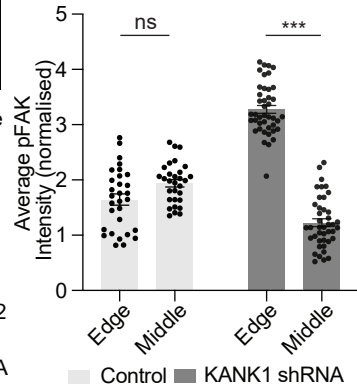

Supplement: Figure S2 [file mmc3.pdf]

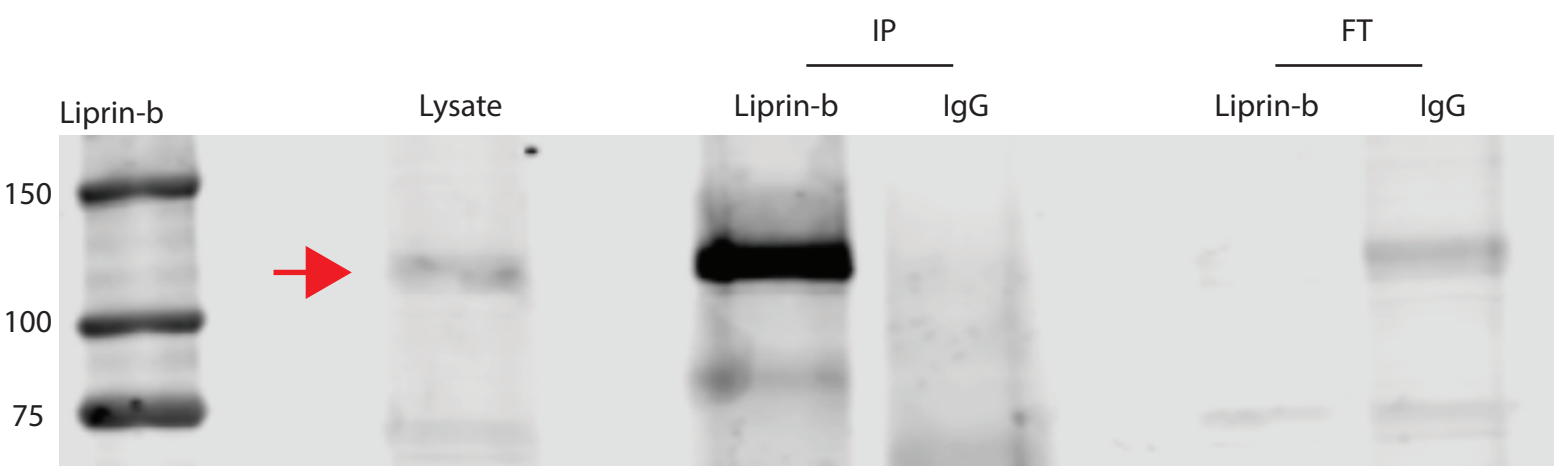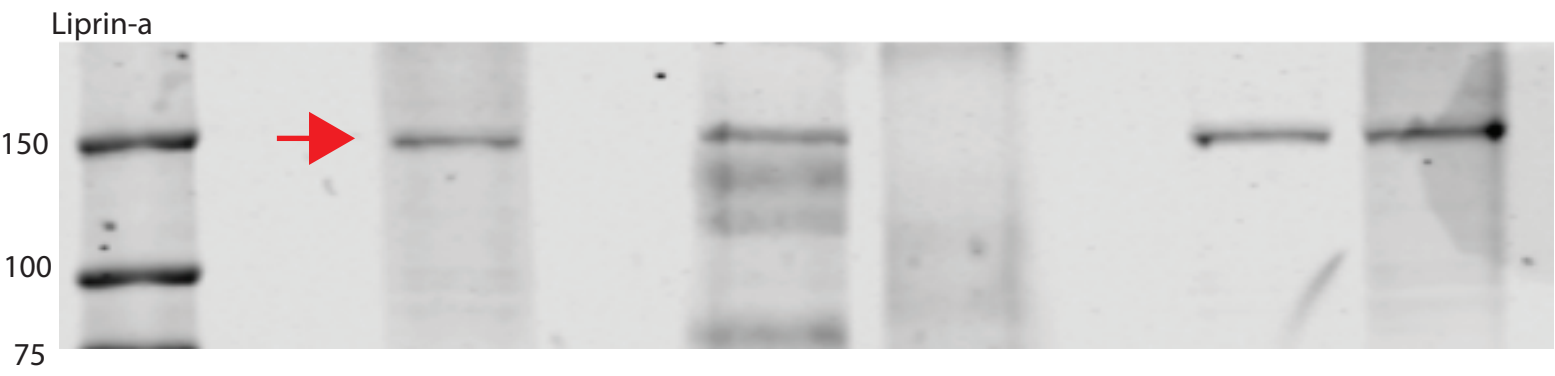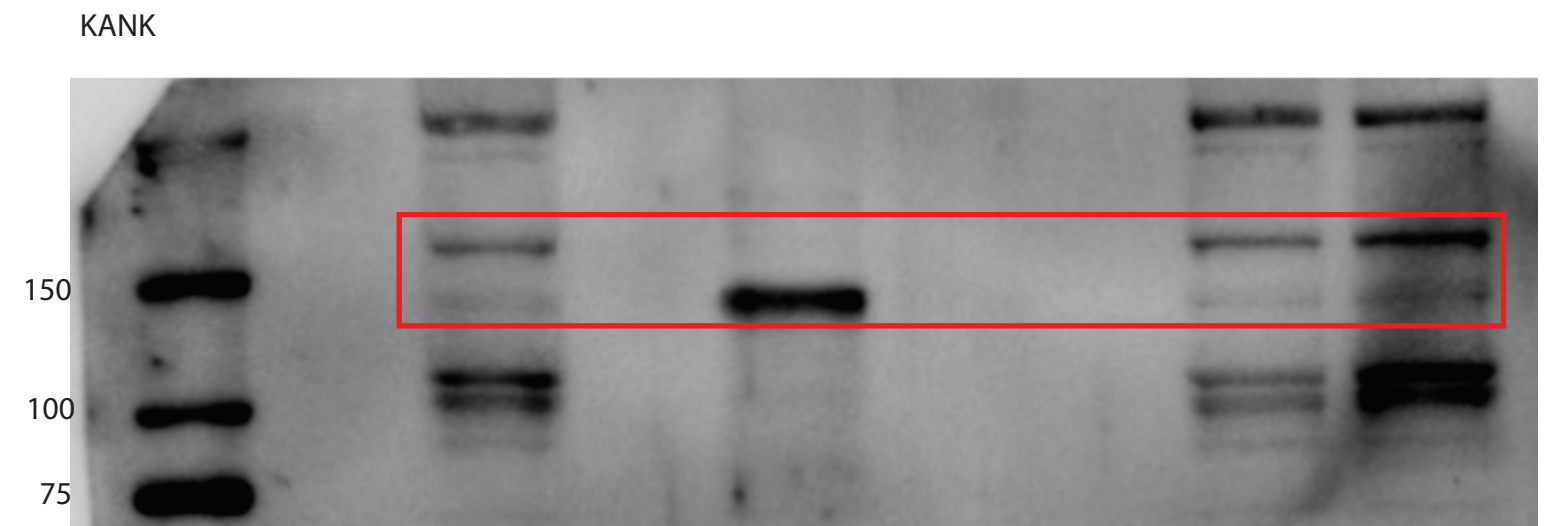

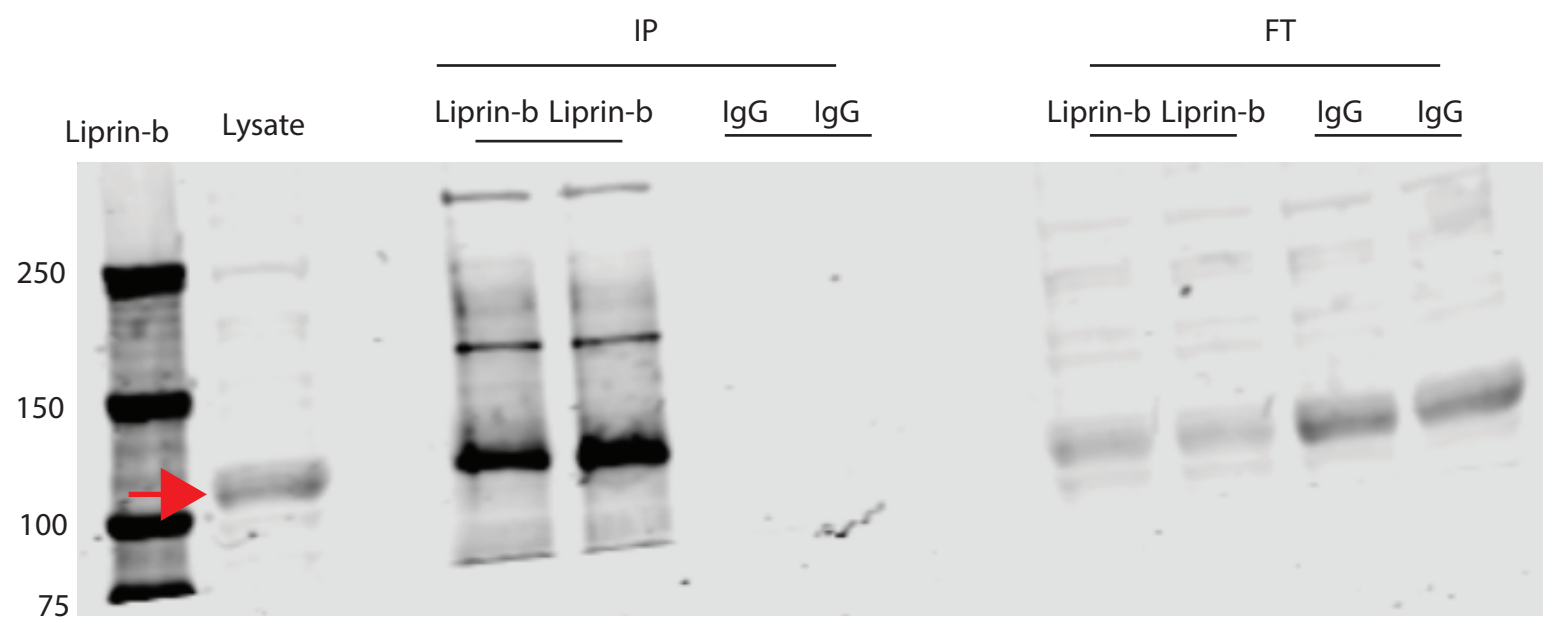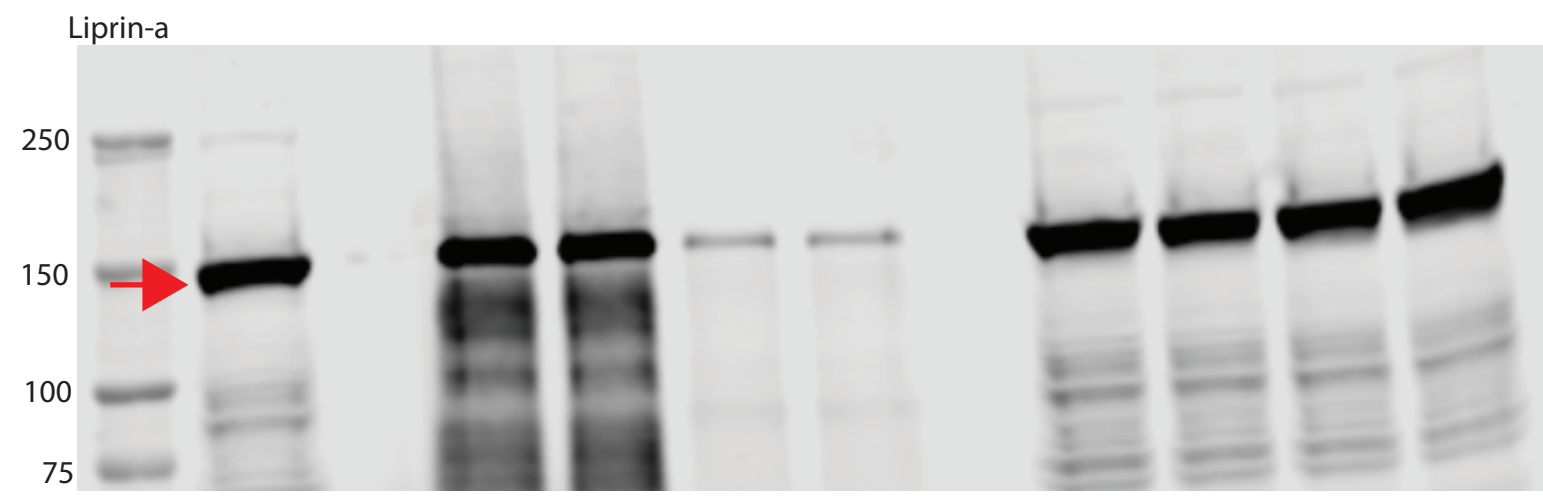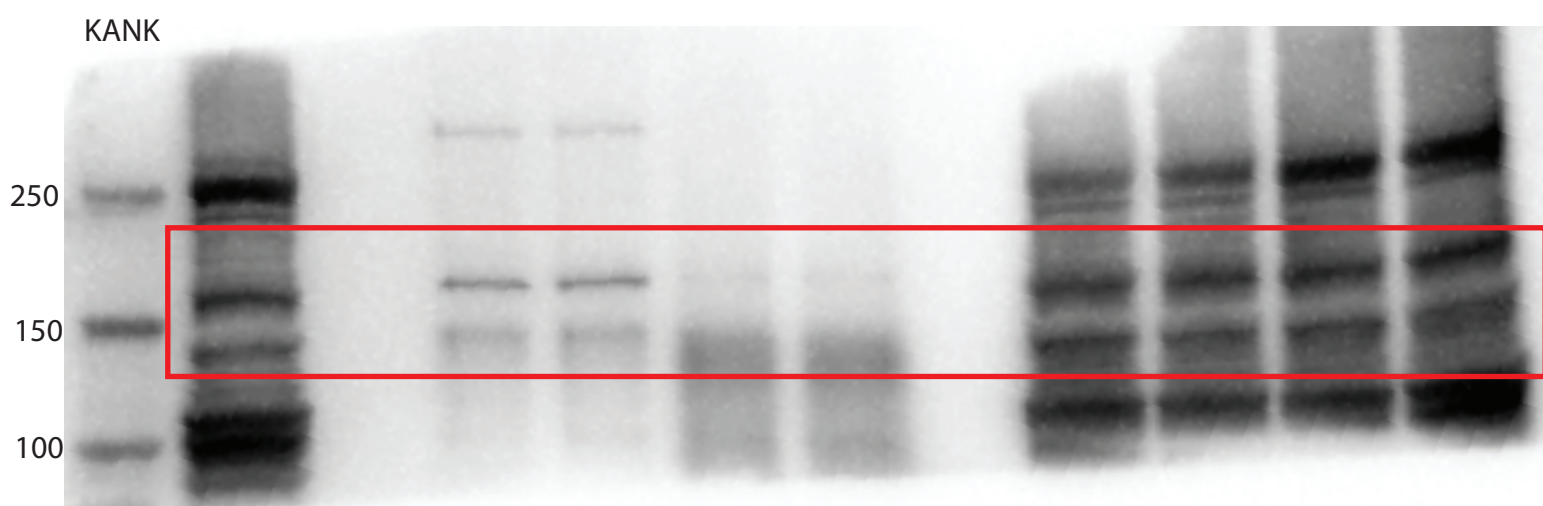

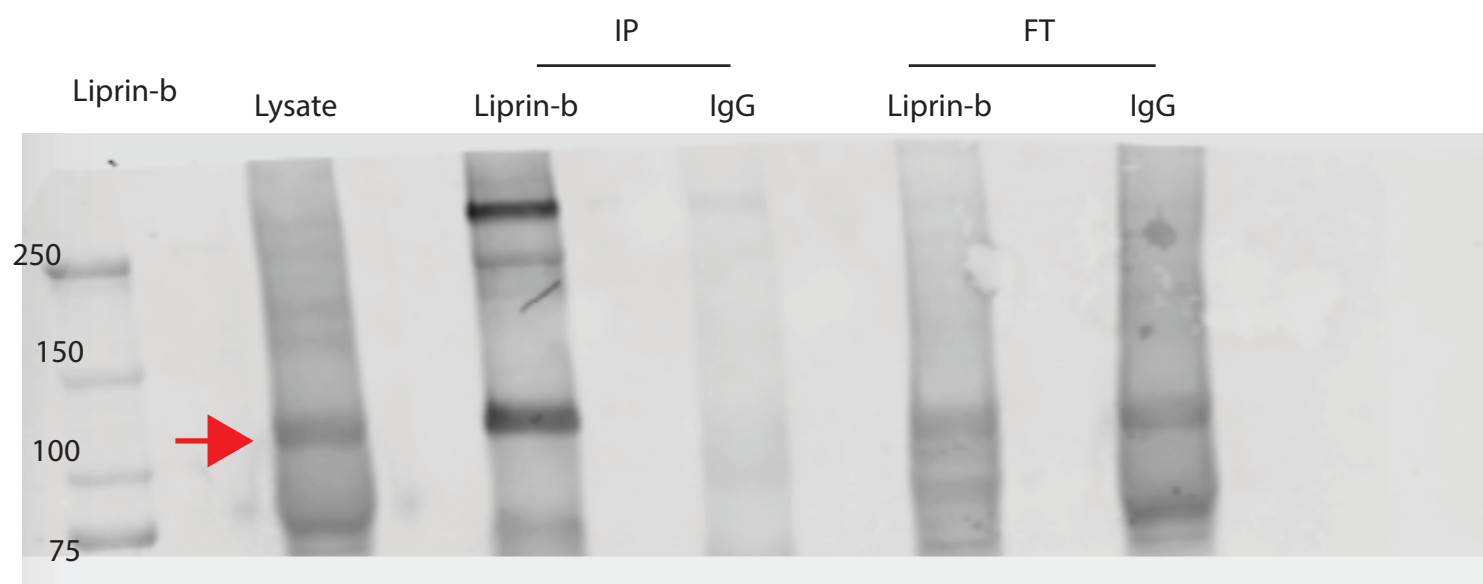

Liprin-a

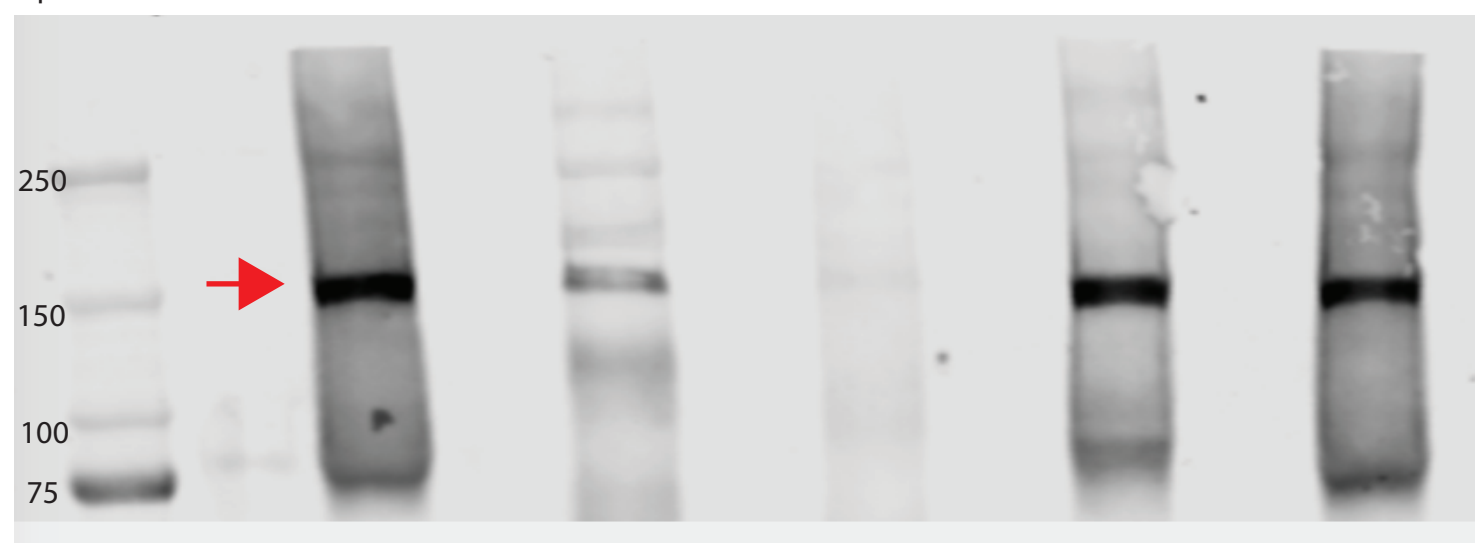

KANK

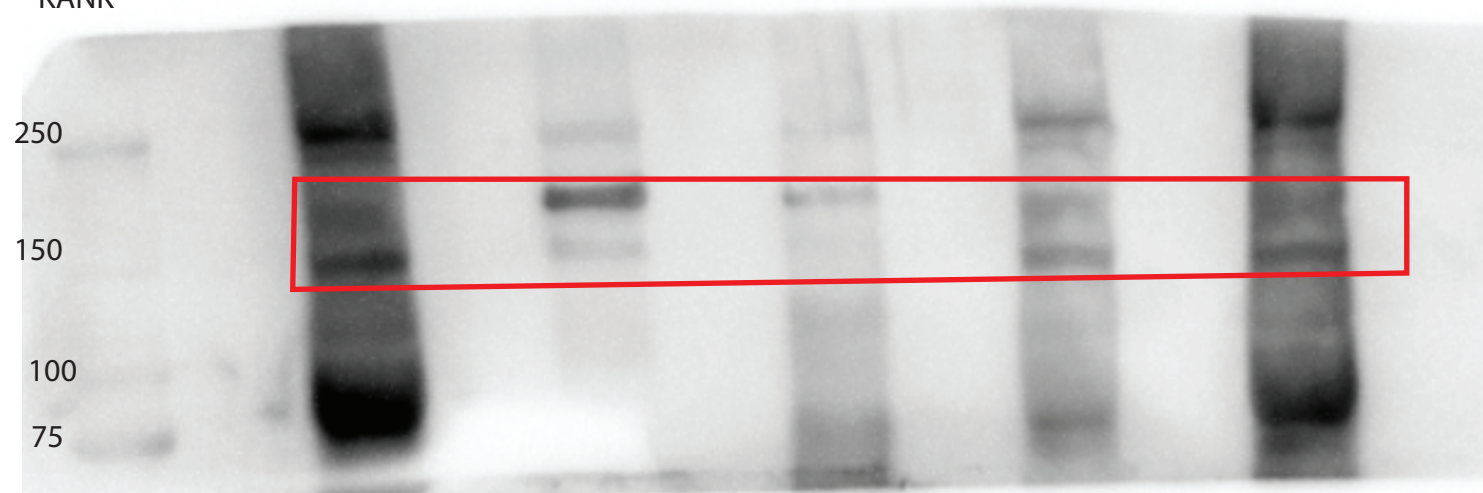

Supplement: Figure S3 [file mmc4.pdf]

# Immunoprecipitation of liprin beta 1 with GFP-liprin-C

Run 1

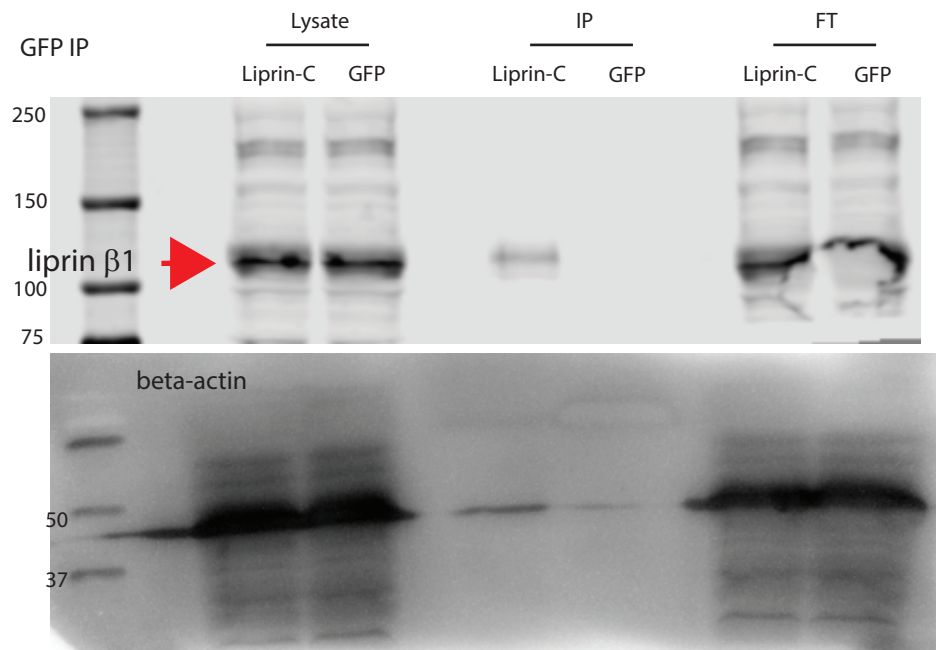

Run 2

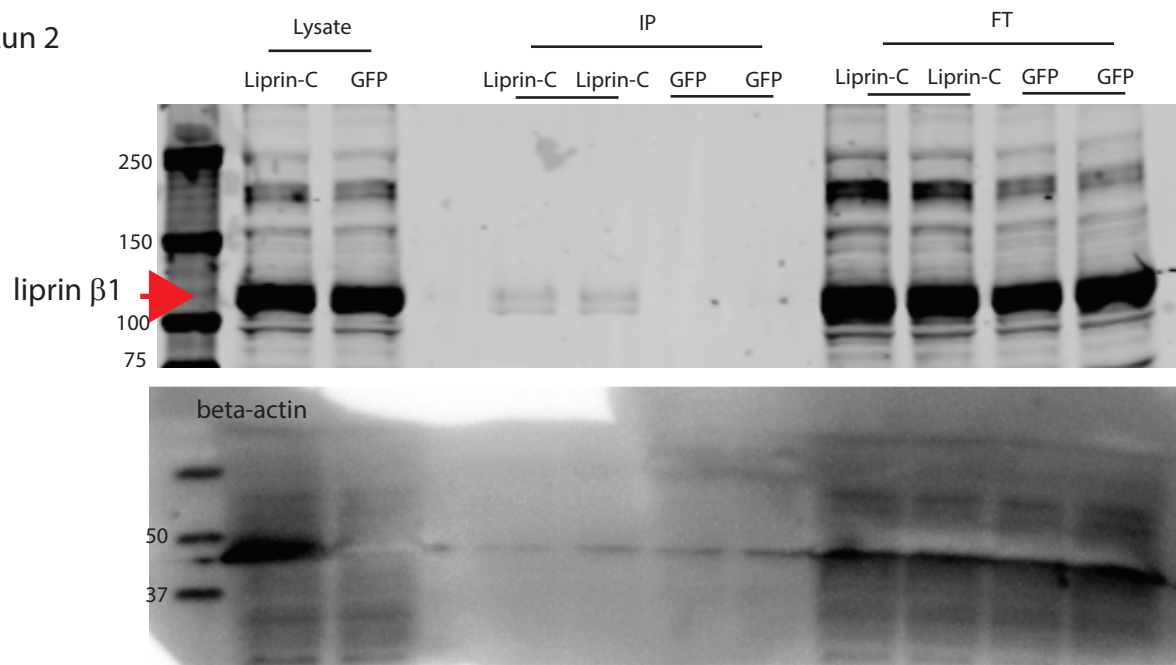

Run 3

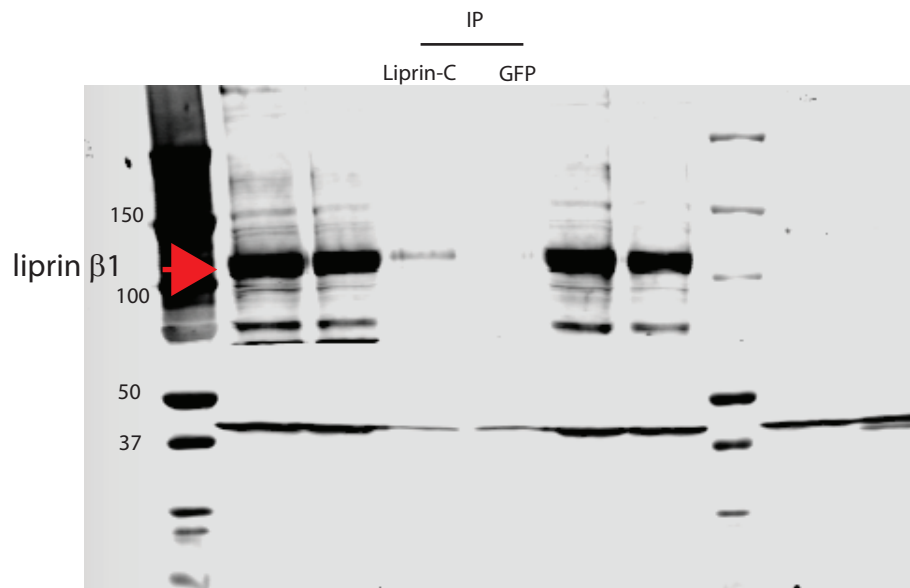

Supplement: Figure S4 [file mmc5.pdf]
